# Transcriptomic analysis of identical twins with different onset ages of adrenoleukodystrophy

**DOI:** 10.3389/fnins.2025.1623285

**Published:** 2025-10-31

**Authors:** Chuhua Fu, Qiuyu Su, Yinglian Chen, Yonghui Zhang, Yan Zhang, Ying Cao, Xinggang Wang, Zhiming Zhen, Chen Liu, Zhao Yang, Changlin Yin, Liang Tan

**Affiliations:** ^1^Department of Neurosurgery, JingMen People’s Hospital, Jingchu University of Technology Affiliated JingMen People’s Hospital, JingMen, Hubei, China; ^2^Department of Neurology, The Affiliated Yongchuan Hospital of Chongqing Medical University, Chongqing, China; ^3^Center of Critical Care Medicine, The First Affiliated Hospital (Southwest Hospital) of Army Medical University, Chongqing, China; ^4^State Key Laboratory of Resource Insects, College of Electronic and Information Engineering, Southwest University, Chongqing, China; ^5^Department of Radiology, Southwest Hospital, Army Medical University (Third Military Medical University), Chongqing, China

**Keywords:** adrenoleukodystrophy, *ABCD1* gene, transcriptomic analysis, identical twins, differentially expressed genes

## Abstract

**Introduction:**

Adrenoleukodystrophy (ALD) is a rare X-linked neurogenetic disease caused by mutations in the ATP-binding cassette subfamily D member 1 (*ABCD1*) gene. Currently, the molecular mechanisms underlying the onset and severity of ALD remain unclear. Therefore, the aim of this study is to identify information on candidate genes associated with the onset and severity of ALD by transcriptome sequencing of whole blood samples from monozygotic twin families with the disease.

**Methods:**

The identification of differentially expressed genes (DEGs), set theory analysis, gene enrichment analysis, and classification statistics of expression trends have been executed to acquire potential candidate genes inducing the onset and severity of ALD in patients. The study cohort comprised eight individuals: two normal children, two pediatric twins with ALD, the twins’ mother, their adult uncle with ALD, the twins’grandmother, and one normal adult.

**Results:**

Five distinct sets of differentially expressed genes (DEGs) were identified using whole blood samples from a family of identical twins with different onset ages and ABCD1 exon 2 deletions. Then, 39 DEGs of A ∩ B ∩ C − D and A ∩ B − D, as well as 425 DEGs of C ∩ E, were considered as genes relating to the onset and severity of ALD. In particular, C4BPA, TPBG, CEP112, CHST15, SMAD1, IL-26, and *LRRC69* have shown more importance than others in ALD onset. Furthermore, KEGG and GO enrichment further suggested the role of Ca^2+^ homeostasis and the plasma membrane in ALD onset and severity. Finally, expression pattern analysis further demonstrated the pivotal role of the selected DEG sets.

**Discussion:**

The information on candidate genes of this study was considered crucial for preliminarily exploring the molecular mechanisms related to the onset and severity of ALD, which offered novel insights and research directions for mitigating and treating the development of ALD.

## 1 Introduction

X-linked adrenoleukodystrophy (ALD) is a rare genetic disease caused by mutations in the ATP-binding cassette subfamily D member 1 (*ABCD1*) gene (Mares Beltran et al., 2024). This gene encodes a peroxisomal membrane protein, which is critical for transporting very long-chain fatty acids (VLCFAs) for beta-oxidation (Buda et al., 2023). Mutations in the *ABCD1* gene disrupt the metabolism of VLCFAs, which leads to damage in the adrenal glands and brain white matter (Launay et al., 2024). The incidence of ALD is approximately one in 15,000 individuals, with various onset times and severity levels (Burton et al., 2022). In males, around 1% are asymptomatic, whereas 31–35% of patients develop ALD symptoms during childhood (at 3–11 years old), and 4–7% of patients show illness characteristics during adolescence (at 11–21 years old) (Turk et al., 2019). Approximately 20% of individuals develop the adult-onset type of adrenomyeloneuropathy (AMN), which typically starts at age 30 (Volmrich et al., 2022). Heterozygous females with mutations are less sensitive to ALD and tend to exhibit symptoms later in life. There are differences in the onset age and severity among patients with ALD, but the molecular mechanisms governing these variations are not fully understood. Understanding these mechanisms is very valuable to aid in delaying the onset and reducing the severity by intervening in patients with *ABCD1* gene mutations to enhance patients’ quality of life. In this study, whole blood samples were obtained from a family with a deletion in exon 2 of the *ABCD1* gene. The samples included homozygous identical twins with the deletion; the twins’ mother and grandmother, who are carriers; a homozygous twin uncle with the deletion; two normal children of similar age to the twins; and one normal adult male as a control. Interestingly, only one of the twin children displayed ALD symptoms, whereas the other twin and their uncle did not show any ALD or AMN symptoms. Transcriptome sequencing analysis of these samples was performed to explore the molecular mechanisms governing the variation in onset time and disease severity among patients with ALD with an exon 2 deletion in the *ABCD1* gene. This study offers new insights and avenues into the management and treatment of ALD.

## 2 Materials and methods

### 2.1 Sample collection and processing

Blood samples (2 + 2 mL) from both the familial patients and the control group were collected using Vacutainer EDTA-K2 anticoagulant tubes (BD 367841) from BD Medical Devices (Shanghai) Co., Ltd. RNA was extracted using TRIZOL lysis, followed by chloroform phase separation, isopropanol precipitation, and 70% ethanol washing. Whole blood samples underwent high-throughput NGS transcriptome sequencing analysis by Shanghai Biotechnology Co., Ltd. to ensure sequencing quality and obtain the sample TPM expression matrix data. Simultaneously, mutations in the *ABCD1* gene were identified by MGI Tech Co., Ltd. via a high-throughput whole-exome sequencing method. Briefly, transcriptome sequencing analysis involves several steps. First, the raw data were quality-controlled using Fastp. Subsequently, the processed reads were aligned to the reference GRCh38 sequence file using Hisat2. The aligned reads are then converted to BAM format using Samtools. Next, gene expression was quantified and transformed into relative expression TPM values using Feature Counts software. For genetic mutation detection, a probe-based next-generation sequencing technique was utilized. Initially, the raw data were quality controlled using Fastp. The quality-controlled reads were aligned to the reference sequence file GRCh37 using BWA. Finally, mutations were analyzed using GATK to obtain variant information. Sample classification and annotation for sequencing are detailed in Table 1. The biochemical and neuropsychological assessment profiles are shown in Supplementary Table 1, which includes behavioral scores and blood lipid-related biochemical indicators to identify the clinical manifestations of ALD. Asymptomatic male patients with ALD and female carriers presented behavioral scores similar to those of normal individuals. However, their lipid-related parameters, such as C24:0 fatty acids and C26:0 fatty acids, were higher than those in the normal control group.

**Table 1:** Detailed sample information of transcriptome sequence.

ID	Class	Sex	Age (years)	Diagnose	Gene type	Note
T1	PT1	Male	8	ALD	Homozygote	Twins
T2	PT1	Male	8	Asymptomatic	Homozygote	Twins
PM	FXX	Female	40	Carrier	Heterozygote	The twins’ mother
PG	FXX	Female	68	AMN	Heterozygote	The twins’ grandmother
CF	NC2	Male	33	Normal	Normal individual	Normal adult
TU	PT2	Male	41	Asymptomatic	Homozygote	The twins’ uncle
C1	NC1	Male	7	Normal	Normal individual	Normal children
C2	NC1	Male	7	Normal	Normal individual	Normal children

Homozygote indicates that the individual’s gene type belongs to a homozygote with ABCD1 exon 2 deficiency. A heterozygote indicates that the individual’s gene type belongs to a heterozygote with ABCD1 exon 2 deficiency. ABCD1 exon 2 deficiency is characterized by a variant c.901_1081del in exon 2 of the gene transcript NM_000033.4. Identification of ABCD1 gene mutations or deletions via second-generation whole-exome sequencing read alignments. For detailed methodological procedures, please refer to the Methods section.

### 2.2 Differential gene screening

After obtaining the gene expression matrix of the samples, five combinations (refer to Table 2) were designed for selection and differential gene screening based on the genotype of the samples and the individuals’ disease status, such as the presence or absence of a disease phenotype. Differential gene screening and the comparison of gene expression levels between the two groups were conducted by using R software (V4.4.1) and code in GitHub.^1^ In cases where there were two sample replicates in each group, genes with an average difference greater than 2-fold and a significance level less than 0.05 were selected as the differential gene set. When there was only one sample replicate in each group, genes with a difference greater than 2-fold and an average deviation or coefficient of variation greater than 0.5 were chosen as differential genes.

**Table 2:** Annotated table of differentially expressed gene sets.

Comparative ways	DEG set names
PT1 group vs. NC1 group	DEGs A
PT1 group vs. FXX group	DEGs B
TU group vs. NC2 group	DEGs C
NC1 group vs. FXX group	DEGs D
T1 vs. T2	DEGs E

DEGs (differentially expressed genes); the PT1 group (comprising samples T1 and T2) represented twins with an ABCD1 gene exon 2 defect. The NC1 group (comprising samples C1 and C2) included samples from normal children. The FXX group (comprising samples PG and PM) represented the twins’ grandmothers (PG) and mothers (PM), respectively. The TU group (sample TU) represented the twins’ uncle with an ABCD1 gene exon 2 defect. The NC2 group (sample CF) comprised normal adults.

The differential gene sets were identified and named as shown in Table 2, and the differentially expressed genes between pairwise groupings were obtained after filtering for extreme values. Subsequently, volcano plots and expression scatter plots were generated using the ggplot2 (V3.5.1) package in R (V4.4.1).

### 2.3 Set theory analysis of differential gene sets

The differential gene sets from each comparing group were subjected to set theory analysis using R software (V4.4.1). DEGs A, B, C, D, and E were examined to identify the gene set A ∩ B ∩ C ∩ E − D, which consists of genes shared among A, B, C, and E but not present in D, and the gene set had served as the candidate gene set for expression differences in whole blood due to the deletion of exon 2 of the *ABCD1* gene. Furthermore, the intersection of DEGs C and E, denoted as C ∩ E, was considered the gene set associated with the onset of ALD in patients.

A Venn diagram and candidate gene expression heatmap were generated using the Venn Diagram (V1.7.3) and pheatmap (V1.0.12) packages in R (V4.4.1). The gene set intersection of DEGs C and E was subjected to Gene Ontology (GO) and Kyoto Encyclopedia of Genes and Genomes (KEGG) enrichment analysis using the David functional annotation tools. The top 10 significantly enriched results, which were ranked by order of *p*-values, were selected for visualization using the ggplot2 (V3.5.1) package in R (V4.4.1).

### 2.4 Analysis of expression trend classification

The control expression means (Cm) of genes in C1 and C2 samples and the expression data of T1, T2, and TU samples were utilized for expression trend pattern classification analysis via using the Mfuzz (V2.64.0) package (Kumar and Mfuzz, 2007) in R (V4.4.1). Subsequently, the distribution quantities of candidate genes from A ∩ B ∩ C − D and C ∩ E were calculated within the corresponding trend classifications and visualized.

## 3 Results

### 3.1 Analysis and statistics of differential gene sets

**Figure 1:**
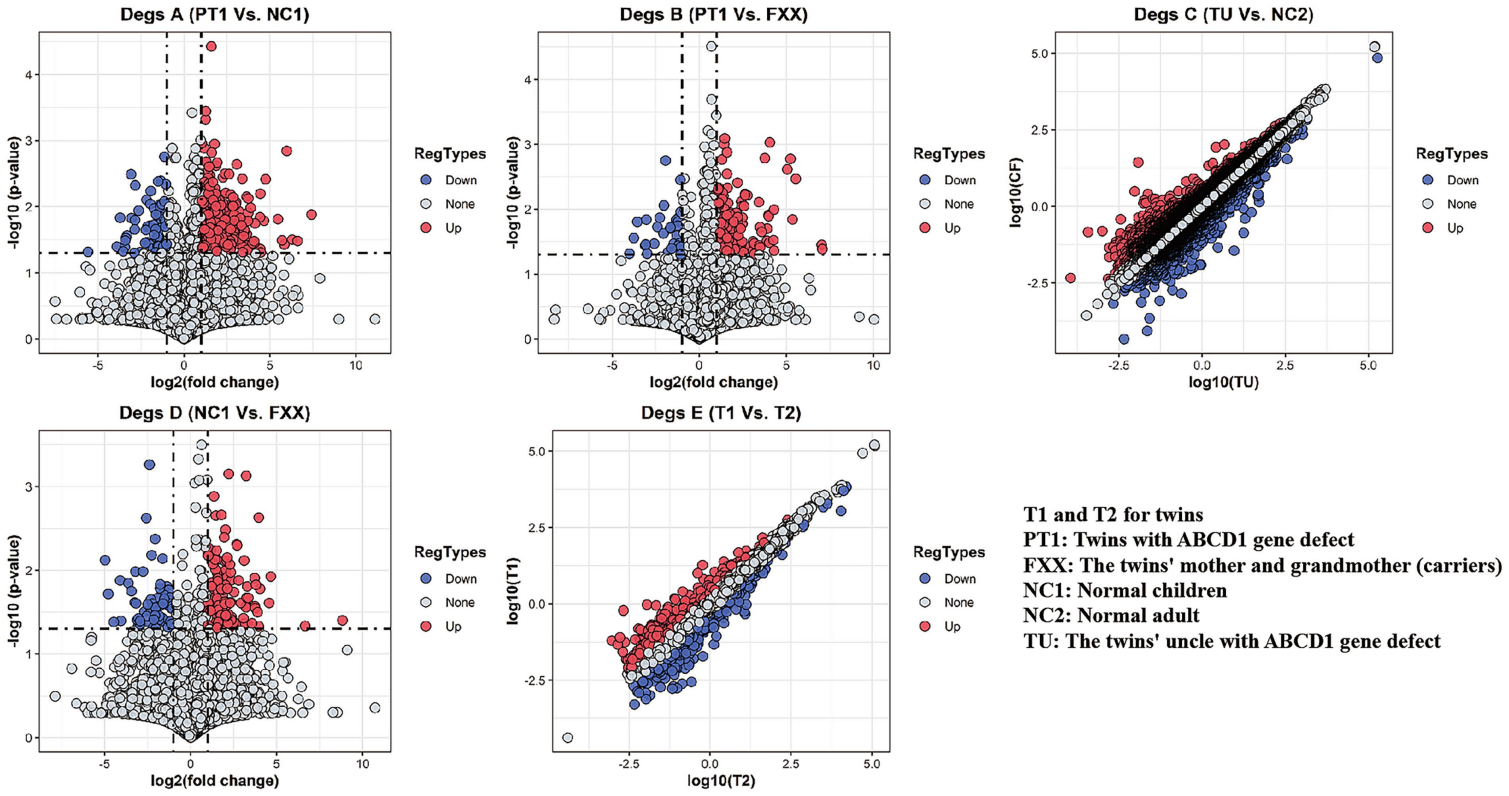
Volcano and scatter plots of differential expression gene sets. The comparison of differential gene sets is shown in Table 2, and the detailed sample information is described in Table 1.

In DEGs A (between PT1 and NC1 class samples), 897 genes were upregulated and 101 genes were downregulated in PT1 compared to NC1. DEGs B (between PT1 and FXX class samples) showed 218 upregulated and 35 downregulated genes in PT1 relative to FXX. Analysis of DEGs C (between TU and NC2 class samples) displayed 1,417 upregulated and 794 downregulated genes in TU compared to NC2. DEGs D (between NC1 and FXX class samples) exhibited 138 upregulated and 78 downregulated genes in NC1 compared to those in FXX. Moreover, DEGs E (between T1 and T2 samples) indicated 636 upregulated and 714 downregulated genes in T1 relative to T2. Figure 1 shows the analytical volcano plot and scatter plots of the differential gene expression of the five sets, which show the corresponding comparison information of the differential gene sets.

### 3.2 Set theory analysis of candidate feature gene sets

**Figure 2:**
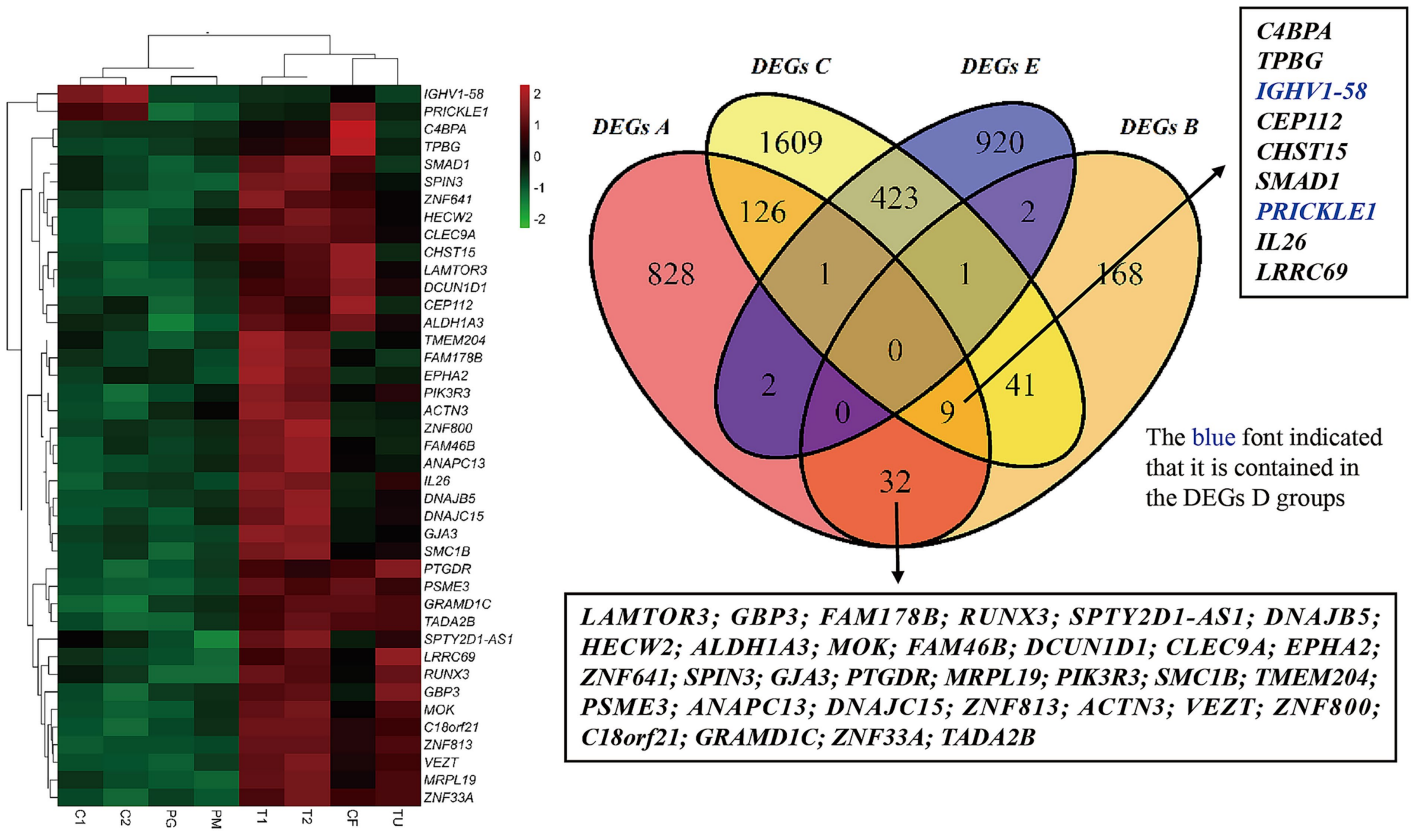
Expression heatmap of 41 candidate genes and Venn diagram of different DEG sets. The left figure shows the expression levels of 41 candidate genes in different samples, where red indicates high gene expression, green indicates low gene expression, and black indicates an excessive color. The rows represented different sample types, and the columns represented 41 candidate genes. In the column labels, C1 and C2 represented samples of normal children; T1 and T2 showed the twins with the *ABCD1* gene exon 2 defect; TU showed the twins’ uncle with the *ABCD1* gene exon 2 defect; PG and PM represented the twins’ grandmother and mother, respectively; and CF represented a normal adult. The 41 candidate genes were selected from the set analysis shown in the right figure, specifically from DEGs A ∩ B ∩ C and DEGs A ∩ B. The right figure is a Venn diagram representing set-based selection, including four differentially expressed gene (DEG) sets: DEGs A, B, C, and E, which were derived from Table 2.

By conducting set theory analysis on the intersections of different DEG sets, it was observed that there were no DEGs that were present in DEGs A, B, C, and E simultaneously while absent in D. However, 7 differentially expressed genes, including *C4BPA, TPBG, CEP112, CHST15, SMAD1, IL-26*, and *LRRC69*, had been shared by DEGs A, B, and C sets but were not present in D. Additionally, 41 genes, including *LAMTOR3, GBP3, FAM178B, RUNX3, SPTY2D1-AS1, DNAJB5, HECW2, ALDH1A3, MOK, FAM46B, DCUN1D1, CLEC9A, EPHA2, ZNF641, SPIN3, GJA3, PTGDR, MRPL19, PIK3R3, SMC1B, TMEM204, PSME3, ANAPC13, DNAJC15, ZNF813, ACTN3, VEZT, ZNF800, C18orf21, GRAMD1C, ZNF33A, TADA2B, C4BPA, TPBG, IGHV1-58, CHST15, SMAD1, PRICKLE1, IL26*, and *LRRC69*, had been shared between DEGs A and B. Among these, *IGHV1-58* and *PRICKLE1* had been found in the differential gene set DEGs D. The intersection of DEGs C and E consisted of 425 genes (Additional Data Table 1). Figure 2 illustrates the Venn diagram of the selected candidate gene sets from the set theory analysis and the sample expression heatmap of 41 genes.

### 3.3 GO and KEGG enrichment analysis of C ∩ E gene set

**Figure 3:**
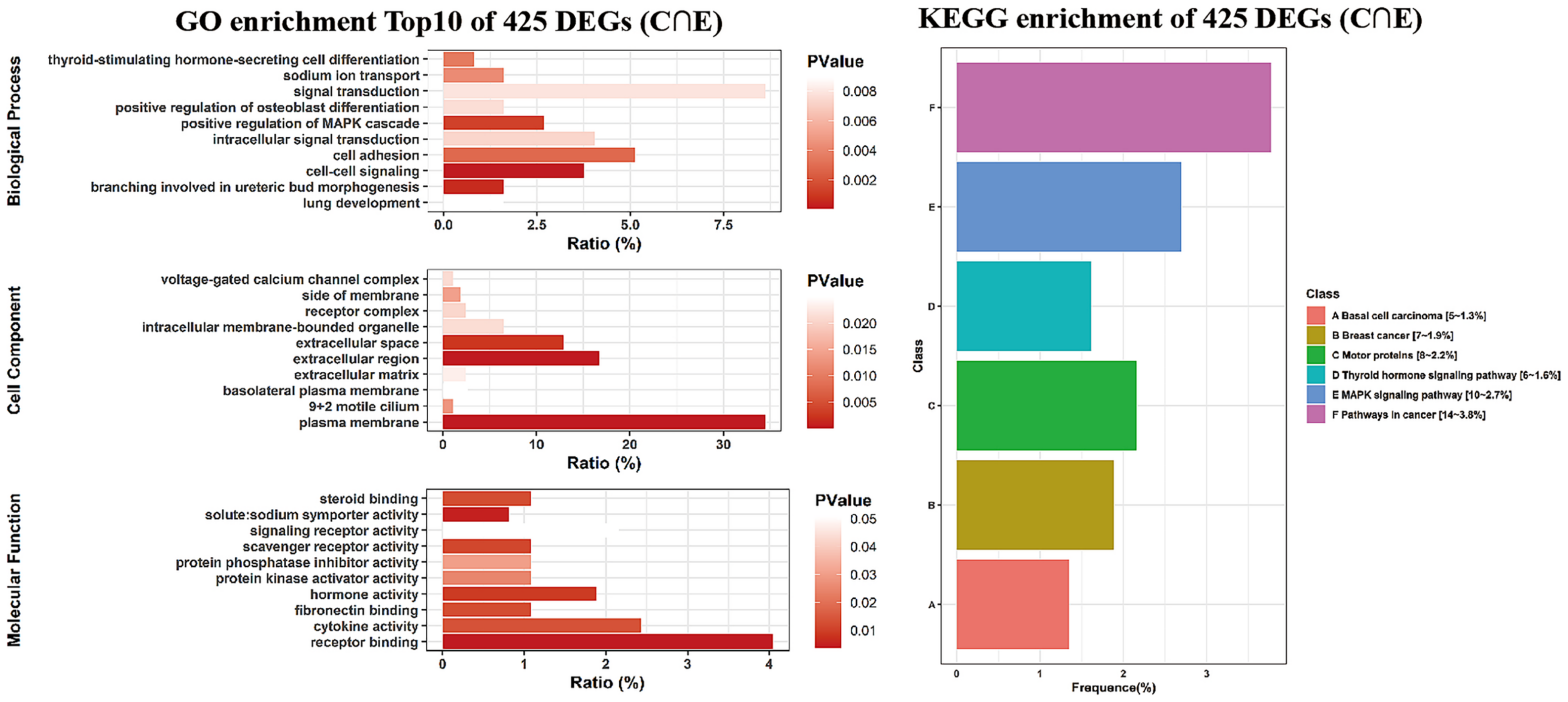
GO and KEGG enrichment plots of intersected genes between DEGs C and D. The GO enrichment plot is shown on the left, and the top 10 results in descending order of *p*-values are displayed. All KEGG enrichment plots are shown on the right side.

GO functional enrichment and KEGG pathway enrichment analyses were performed on the differential gene set C ∩ E. The results (Figure 3) showed significant rankings of the top 10 biological processes, cellular components, and molecular functions in GO functional enrichment.

The top biological processes included cell–cell signaling (GO:0007267), branching involved in ureteric bud morphogenesis (GO:0001658), positive regulation of the MAPK cascade (GO:0043410), cell adhesion (GO:0007155), thyroid-stimulating hormone-secreting cell differentiation (GO:0060129), sodium ion transport (GO:0006814), intracellular signal transduction (GO:0035556), positive regulation of osteoblast differentiation (GO:0045669), signal transduction (GO:0007165), and lung development (GO:0030324).

Enriched cellular components encompassed the plasma membrane (GO:0005886), extracellular region (GO:0005576), receptor complex (GO:0043235), intracellular membrane-bounded organelle (GO:0043231), extracellular matrix (GO:0031012), and basolateral plasma membrane (GO:0016323).

Additionally, enriched molecular functions comprised receptor binding (GO:0005102), solute sodium symporter activity (GO:0015370), hormone activity (GO:0005179), scavenger receptor activity (GO:0005044), fibronectin binding (GO:0001968), steroid binding (GO:0005496), cytokine activity (GO:0005125), protein kinase activator activity (GO:0030295), protein phosphatase inhibitor activity (GO:0004864), and signaling receptor activity (GO:0038023).

KEGG pathway enrichment was primarily observed in pathways such as basal cell carcinoma (hsa05217), breast cancer (hsa05224), motor proteins (hsa04814), and the thyroid hormone signaling pathway (hsa04919).

### 3.4 Gene expression trend clustering analysis

**Figure 4:**
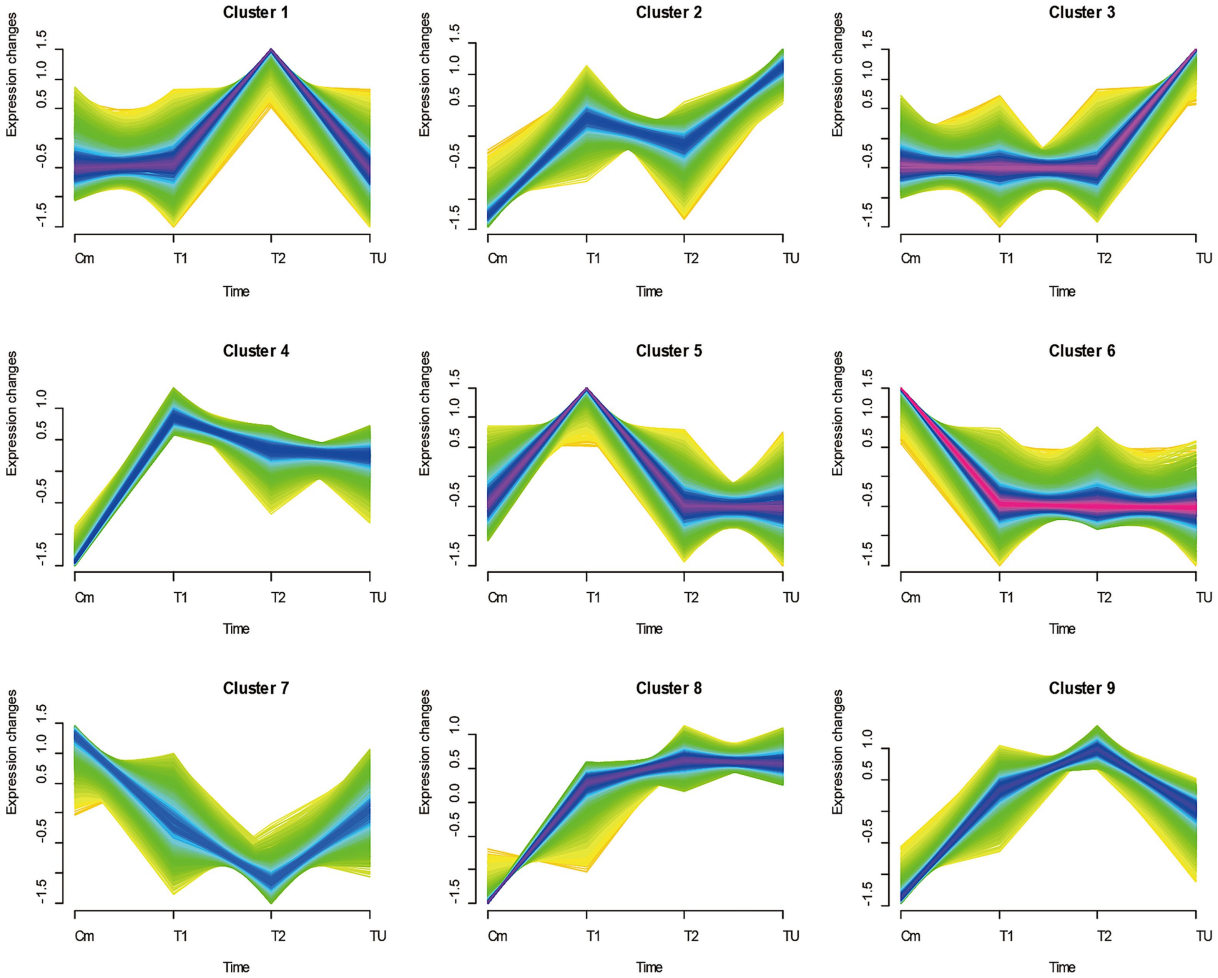
Gene expression trend clustering plots. An expression tendency analysis was conducted on the normal children control group (with mean expression Cm), symptomatic ALD twin T1, asymptomatic ALD twin T2, and asymptomatic ALD uncle (sample TU). The expression trends displayed the nine aforementioned expression patterns.

**Figure 5:**
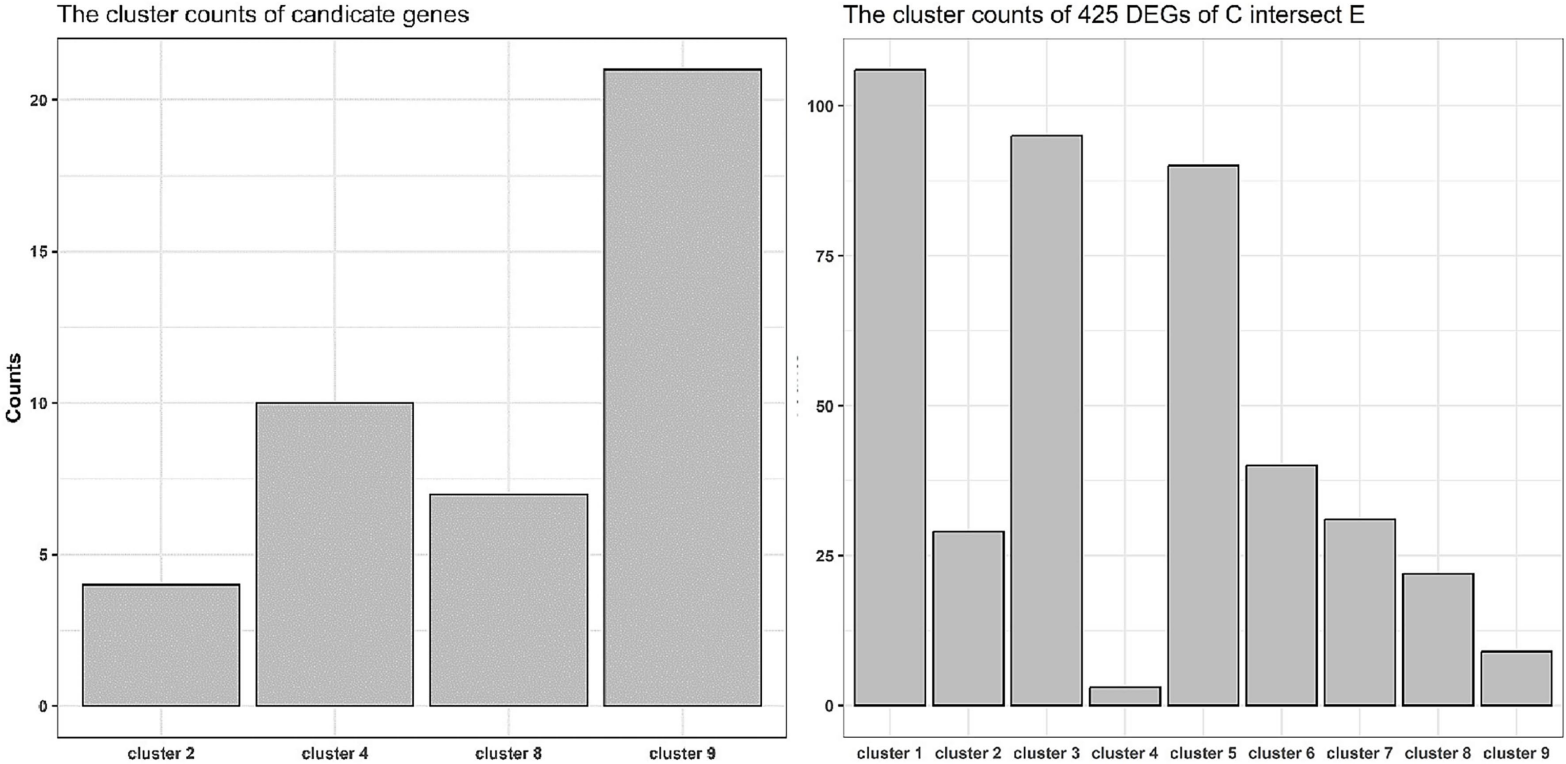
Trend distribution of candidate genes and intersected genes between DEGs C and D. The right panel presented an intersection analysis of the 41 candidate genes with the nine gene sets of expression tendencies defined in Figure 4. This analysis revealed enrichment within tendency categories 2, 4, 8, and 9. The left panel displayed the enrichment profile of the 425 genes from the DEGs C ∩ E set across the same expression tendency categories. In both plots, the x-axis represents the different expression tendency categories, and the y-axis corresponds to the number of enriched genes.

Trend clustering analysis was performed using the gene expression mean values of normal children, twins, and the twins’ uncle samples. The results showed nine types of expression trend patterns (Figure 4). Among the candidate genes from A ∩ B − D, the numbers relating to the 2nd, 4th, 8th, and 9th trend types were 2, 10, 6, and 21, respectively (Figure 5, left panel). For the C ∩ E genes, the numbers relating to the 1st to 9th trend types were 106, 29, 95, 3, 90, 44, 31, 22, and 9 genes, respectively (Figure 5, right panel). Notably, genes such as *LRRC69* and *PTGDR* belonged to the 2nd trend type in the A ∩ B − D gene set, whereas genes such as *CEP112, IL26, RUNX3, ALDH1A3, EPHA2, ZNF641, PIK3R3, TMEM204, PSME3, and C18orf21* were part of the 4th trend type.

In the C ∩ E gene set, genes such as *PRSS53, AMH, SDHD, HOXB7, GALNT8, CLIC6, ZNF24, LURAP1L, SLC13A3, COMT, PAQR3, ZBED6, FOXD4, CACNA2D2, GOLT1B, TRAV17, PCBP4, MGAT5, ITIH2, SHF, CRHBP, CACNB3, GZMK, FKBP10, AK8, ARHGEF25, EBF4, ATP1B2*, and *TBC1D29* were associated with the 2nd trend type, whereas *CYYR1, ANGPTL1*, and *DNAJC28* were linked to the 4th trend type. A more detailed classification of genes can be found in Supplementary Table 2.

## 4 Discussion

The clinical manifestation of ALD does not correlate with genotype; however, both disease severity and age of onset are highly variable among patients (Campopiano et al., 2021; Zemanova et al., 2021). Currently, the molecular mechanisms underlying the appearance of pre-symptomatic indicators or the induction of symptomatic phenotypes in ALD remain unclear. In this study, transcriptomic sequencing analysis was conducted on whole blood samples from individuals with different genotypes and ALD phenotypes within the same family lineage. This study aims to lay the foundation for identifying pre-symptomatic indicators of ALD onset and unravel the molecular mechanisms associated with the severity and onset age of patients with ALD.

### 4.1 DEGs of A ∩ B ∩ C − D and A ∩ B − D were considered as potential candidate genes for inducing the onset of ALD in patients

Based on the pathogenic and nonpathogenic genotypes as well as onset and non-onset states, five different combinations of differential gene sets were designed and are shown in Table 2. First, A represented DEGs between twin patients with ALD and normal genotype children, which reflected the situation caused by the deletion of exon 2 of *ABCD1* but was also constituted by individual genetic differences. Subsequently, B represented DEGs between homozygous twins with a deletion in exon 2 of *ABCD1* and heterozygous carriers, who are the twins’ mother and grandmother, and also encompassed genes related to gender and age differences. Meanwhile, C represented DEGs between the twins’ uncle with a deletion in exon 2 of *ABCD1* and a normal adult male, which included individual differential genes. Notably, D represented DEGs between normal children and the mothers and grandmothers of twins, which could be used for screening genes related to individual genetic, gender, and age differences in an exclusionary way.

The *ABCD1* gene is located in the peroxisomal membrane and plays a primary role in transporting VLCFAs for beta-oxidation. The second exon of the *ABCD1* gene encodes the range from 301 to 361 of amino acid residues and is located in the fifth transmembrane domain of the *ABCD1* gene (Zuo and Chen, 2024). Abnormal function of the *ABCD1* gene leads to the accumulation of VLCFAs to induce cell apoptosis or necrosis through various factors such as destabilizing the cytoplasmic membrane (Basta and Pandya, 2024), affecting the proper folding of endoplasmic reticulum proteins (Wanders et al., 2023), impacting mitochondrial ATP synthase efficiency (Parasar et al., 2024), increasing oxidative stress sensitivity, and elevating mitochondrial DNA damage caused by reactive oxygen species in cells (Marchetti et al., 2018). In summary, the filtered DEGs by the A ∩ B ∩ C − D set calculation included *C4BPA, TPBG, CEP112, CHST15, SMAD1, IL-26,* and *LRRC69*, which had been selected as critical genes for patients with a deletion in exon 2 of *ABCD1*. In previous reports, *SMAD1* had been shown to correlate with diseases of the nervous system and the development of neural stem cells (Okur et al., 2024; Wolf et al., 2023; Yang et al., 2022). ALD predominantly affects the nervous system, leading to symptoms such as unsteady gait and cognitive decline. Therefore, *SMAD1* may be linked to ALD onset. Nevertheless, this hypothesis requires confirmation through additional functional experiments, such as observing changes in cognitive and learning abilities after knocking out the *SMAD1* gene in ALD animal models. Additionally, *TPBG* and *CEP112* have been shown to be related to glioblastoma and Alzheimer’s disease (Zhang et al., 2021; Homann et al., 2022). Particularly, the higher content of immune cell components such as leukocytes and macrophages in whole-blood samples will accelerate the accumulation of VLCFAs in the cytoplasm because of macrophage phagocytosis of cell debris (Zierfuss et al., 2022). Furthermore, *C4BPA* and *IL-26* are immune-related complement system genes and inflammation-related factors, respectively, and it has been suggested that the deletion of exon 2 of *ABCD1* could affect the nonspecific complement system or immune-inflammatory system due to demyelination of neurons (Gupta et al., 2021) or metabolic disorder of immune factors (Swaminathan et al., 2022) in some patients with ALD. Similarly, it was reported that saturated VLCFAs provoked pro-inflammatory responses through chemokine release in primary macrophages (Zierfuss et al., 2022). The *TPBG* and *CEP112* genes, which are implicated in neurological disorders, may offer insights into the onset of ALD. However, their direct association with ALD warrants further experimental validation. Moreover, immune effects mediated by *C4BPA* and *IL-26* through mechanisms including macrophage phagocytosis and inflammatory responses can contribute to both the severity of ALD and its associated neurological impairment, which contributes to the understanding of the pathogenesis of ALD. Therefore, the results of the set theory analysis on A ∩ B ∩ C − D have been provided as a reference for studying the molecular mechanisms relating to the onset age and severity of ALD, as well as for screening ALD disease markers. However, A ∩ B − D also holds significance as a reference result because of the single sample in group C, which made it difficult to obtain an accurate set of DEGs.

### 4.2 KEGG and GO enrichments of shared DEGs between C and E sets for assessing the importance of differential genes

In this study, E was displayed as DEGs between twin patients with ALD, which represented the different onset times of ALD twins with homozygous deletion of exon 2 of the *ABCD1* gene. Hence, the shared DEGs of C and E constituted pivotal genes related to the onset of ALD. To further explore the biological mechanism and provide more evidence on the onset time of ALD, KEGG and GO enrichment analyses were performed and revealed that DEGs are mainly enriched in functions related to the plasma membrane, cell signaling, and cellular communication. In particular, the transport of calcium ions in biological processes is consistent with the results of previous studies, which showed that an increase in long-chain fatty acid content in the cytoplasm disrupts calcium ion homeostasis (Van Haren et al., 2023; Hein et al., 2008). Meanwhile, in a recent study, it was shown that increasing vitamin D levels would increase cerebral blood flow in boys with ALD (Zhao et al., 2023), which further provides evidence on the onset of ALD for the importance of shared DEGs of C and E. Therefore, the above results also suggest the importance of plasma membrane and intercellular signal transduction in the pathogenesis or onset of ALD because the accumulation of unsaturated VLCFAs in the cytoplasm will contribute to hydroxycholesterol synthesis, which ultimately damages the stability of the plasma membrane, especially in neuronal cells, to induce demyelination (Jang et al., 2016; Dong et al., 2023). However, the detailed biological mechanism needs more experimental evidence, although KEGG and GO enrichment results provided corresponding suggestions, and the results merely serve as reference data for the readers.

### 4.3 The analysis of temporal expression trends showed the significance of candidate genes in the onset age and severity of ALD

To further filter out potential candidate genes and understand the expression patterns of the candidate genes related to ALD onset, expression trend clustering analysis was executed using all genes of twins, normal children, and the twins’ uncle samples. The average expression value (Cm) of normal control children was used as the starting point. Both twins have a deletion in exon 2 of the *ABCD1* gene, but T2 shows asymptomatic manifestations, and T1 shows ALD manifestations. Meanwhile, the TU sample constituted the same genotype but still consistently remained asymptomatic. According to theoretical expectations, candidate genes in the T1 and T2 samples should exhibit expression differences compared to the Cm group. Therefore, genes with the 1st, 2nd, 4th, 5th, 7th, and 8th trend classes were maintained using theoretical rules. Additionally, the expression difference between T2 and the reference starting point Cm should be smaller than that between T1 and Cm due to the asymptomatic nature of T2, so that the 2nd, 4th, and 5th expressional trends were satisfactory. Almost all the shared DEGs between the A and B sets were in line with the second and fourth expression trends, which further hinted at the criticality of screened candidates for the development of ALD. Among the genes in set A ∩ B ∩ C − D, *LRRC69*, *CEP112*, and *IL26* belonged to the second and fourth expression trends, which further emphasized their importance in the progression of ALD. Furthermore, in the 5th class, the gene expression of T1 was higher compared to other asymptomatic individuals with the same expression level, indicating a direct association between the 5th class genes and the onset of ALD. In the DEG set of C ∩ E, 90 of the 425 genes belonged to the 5th class of genes and showed a relationship with ALD onset. However, DEGs of A ∩ B − D were not found to align with the 5th trend genes; therefore, DEGs of A ∩ B − D mainly represented the effect of gene expression caused by gene defects rather than the effect of ALD onset. Notably, the DEGs of C ∩ E also had 106 and 95 genes involved in the 1st and 3rd classes, respectively, which suggested other expressional rules relating to the onset of ALD. Genes were categorized with similar expression patterns to explore the dynamic patterns of candidate genes in the progression of ALD to further confirm highly correlated candidate genes and provide novel insights for potential expression rules related to the onset of ALD. However, the actual expression patterns of genes must be considered because predictive classification is based on mathematical algorithms (Kumar and Mfuzz, 2007).

### 4.4 Limitations

This study employed transcriptome sequencing to conduct a preliminary investigation of the molecular mechanisms of ALD onset. The following three main limitations of this study should be highlighted: 1. Incomplete Mechanistic Validation: Although the twin case suggests a role for exon 2 deletion in *ABCD1*, the ALD pathogenic site mainly occurs in the brain, adrenal gland, and testis (Launay et al., 2024). Therefore, further experimental support through quantitative PCR, Western blot, and gene overexpression or knockdown assays in relevant tissue cells or animal models is required. 2. Inherent Sample Size Constraints: The limited sample size inherent to rare disease research is still unable to meet conventional statistical testing standards, including requirements for replicates and false-positive controls. It is possible that no candidate genes met the threshold for significance when applying significance corrections to small sample sizes. Therefore, it is essential to incorporate more ALD patient samples with identical mutations in future studies to further validate these conclusions. 3. Unaddressed Clinical Heterogeneity: Considering the variation in disease severity associated with different mutation sites, our current sample type is insufficient for a robust analysis of this genotype–phenotype correlation, highlighting the need for future studies to incorporate more patients to substantiate these findings.

In summary, according to the theoretical perspective, the gene set A ∩ B ∩ C − D can be further considered as an induced gene set for the onset of ALD, whereas the genes in set C ∩ E can be inferred as an execution gene set after the onset of ALD. Further experimental studies are needed to validate these inferences because of the lack of similar research patterns and the rarity of cases. A limited sample size can only provide preliminary insights into the molecular mechanisms underlying the onset of ALD. Notably, because of the limited sample size inherent to rare disease research and the current lack of functional validation experiments, further studies with larger patient cohorts as well as animal or cell-based models are required to validate the role of the identified genes in ALD pathogenesis.

## 5 Conclusion

In the present study, transcriptome sequencing analysis was conducted using whole blood samples from a twin family with an *ABCD1* gene exon 2 deletion genotype. Five DEG sets had been screened, and set theory analysis, gene enrichment, and expression trend classification statistics had been performed to identify potential candidate genes inducing the onset of ALD in patients. The DEG sets of A ∩ B ∩ C − D and A ∩ B − D had been filtered out, considering them as genes for the onset of ALD, whereas the DEG set of C ∩ E was considered as the pathological effect gene set for ALD. These results provide reference data for exploring the molecular mechanisms related to the onset age and severity of ALD.

## Data Availability

The datasets presented in this study can be found in online repositories. The names of the repository/repositories and accession number(s) can be found in the article/Supplementary material.

## Footnotes

^1^ https://github.com/YLCHEN1992/ALD_paper
