# Supplementary material for: Transcriptomic analysis of identical twins with different onset ages of adrenoleukodystrophy
Source: Front Neurosci. 2025 Oct 31;19:1623285. doi: 10.3389/fnins.2025.1623285 (PMC12615386; doi:10.3389/fnins.2025.1623285)
Supplement: Supplementary file 1 [file Table_1.DOCX]

| Supplementary Table 1. Biochemical and Neuropsychological Assessment Profiles | | | | | | | | |
| --- | --- | --- | --- | --- | --- | --- | --- | --- |
| ID | T1 | T2 | PM | PG | CF | TU | C1 | C2 |
| TG (mmol/L) | 0.79 | 1.38 | 0.81 | 3.74 | - | 0.9 | - | - |
| CHOL (mmol/L) | 4.19 | 4.22 | 4.8 | 3.74 | - | 4.36 | - | - |
| HDL (mmol/L) | 2.04 | 1.84 | 1.75 | 1.13 | - | 1.2 | - | - |
| LDL (mmol/L) | 2.05 | 2.09 | 2.81 | 1.83 | - | 2.85 | - | - |
| PYR (μmol/L) | 19.2 | 58.1 | 59.3 | 97.5 | - | 39.7 | - | - |
| C22:0 (μg/mL) | 43.6 | 42.6 | 53 | 38.8 | - | 49.3 | - | - |
| C24:0 (μg/mL) | 42.9 | 43.2 | 34.6 | 35.1 | - | 54.2 | - | - |
| C26:0 (μg/mL) | 1.74 | 1.9 | 0.82 | 1.55 | - | 2.44 | - | - |
| C24:0/C22:0 | 0.98 | 1.01 | 0.65 | 0.9 | - | 1.1 | - | - |
| C26:0/C22:0 | 0.04 | 0.045 | 0.015 | 0.04 | - | 0.049 | - | - |
| TNF-α (pg/mL) | 11.2 | 9.2 | 5.65 | 5.09 | - | 9.89 | - | - |
| MoCA | - | 14 | 25 | 13 | - | 26 | - | - |
| DST | - | 10 | 13 | 9 | - | 12 | - | - |
| RSPM | - | 102 | 90 | - | - | 93 | - | - |
| STAI-S | - | - | 57 | 77 | - | 53 | - | - |
| STAI-T | - | - | 46 | 65 | - | 47 | - | - |
| SPPB | 10 | 11 | 12 | 8 | - | 12 | - | - |

Abbreviations: TG, triglycerides (reference range: 0.00-2.30 mmol/L); CHOL, total cholesterol (reference range: 0.00-5.60 mmol/L); HDL, high-density lipoprotein (reference range: Male > 0.90 mmol/L, Female > 1.15 mmol/L); LDL, low-density lipoprotein (reference range: 0.00-4.11 mmol/L); PYR, pyruvate (reference range: 20.0-100.0 μmol/L); C22:0, docosanoic acid (reference range: ≤ 96.3 μg/mL); C24:0, tetracosanoic acid (reference range: ≤ 91.4 μg/mL); C24:0/C22:0, ratio of tetracosanoic acid to docosanoic acid (reference range: ≤ 1.39); C26:0, hexacosanoic acid (reference range: ≤ 1.30 μg/mL); C26:0/C22:0, ratio of hexacosanoic acid to docosanoic acid (reference range: ≤ 0.023); TNF-α, tumor necrosis factor-alpha (reference range: ≤ 8.1 pg/mL); MoCA, Montreal Cognitive Assessment (Version 7.1; score > 25 considered normal); DST, Digit Symbol Substitution Test (Wechsler Adult Intelligence Scale, Fourth Edition: Administration and Scoring Manual, San Antonio, 2008; reference score range: 9-11); RSPM, Raven's Standard Progressive Matrices (The Standardization Study of the Chinese Raven's Progressive Matrices Test conducted at Beijing Normal University, 1989; score > 90 considered normal); SPPB, Short Physical Performance Battery (score > 11 considered normal); STAI-S, State Anxiety Inventory (reference score range: Male 30.82-48.6, Female 30.52-47.42); STAI-T, Trait Anxiety Inventory (reference score range: Male 33.37-48.85, Female 33.77-48.85). All values in parentheses denote the reference ranges for the healthy control group. Abbreviations and reference values are presented consistently throughout.
